# Signatures of post-zygotic structural genetic aberrations in the cells of histologically normal breast tissue that can predispose to sporadic breast cancer

**DOI:** 10.1101/gr.187823.114

**Published:** 2015-10

**Authors:** Lars A. Forsberg, Chiara Rasi, Gyula Pekar, Hanna Davies, Arkadiusz Piotrowski, Devin Absher, Hamid Reza Razzaghian, Aleksandra Ambicka, Krzysztof Halaszka, Marcin Przewoźnik, Anna Kruczak, Geeta Mandava, Saichand Pasupulati, Julia Hacker, K. Reddy Prakash, Ravi Chandra Dasari, Joey Lau, Nelly Penagos-Tafurt, Helena M. Olofsson, Gunilla Hallberg, Piotr Skotnicki, Jerzy Mituś, Jaroslaw Skokowski, Michal Jankowski, Ewa Śrutek, Wojciech Zegarski, Eva Tiensuu Janson, Janusz Ryś, Tibor Tot, Jan P. Dumanski

**Affiliations:** 1 Department of Immunology, Genetics and Pathology and SciLifeLab, Uppsala University, 715 85 Uppsala, Sweden;; 2 Department of Pathology, Central Hospital Falun, 791 82 Falun, Sweden;; 3 Department of Biology and Pharmaceutical Botany, Medical University of Gdansk, 80-416 Gdansk, Poland;; 4 HudsonAlpha Institute for Biotechnology, Huntsville, Alabama 35806, USA;; 5 Centre of Oncology, Maria Sklodowska-Curie Memorial Institute, Kraków Branch, 31-115 Kraków, Poland;; 6 Department of Medical Cell Biology, Uppsala University, 751 23 Uppsala, Sweden;; 7 Department of Medical Sciences, Uppsala University, 751 85 Uppsala, Sweden;; 8 Department of Women's and Children's Health, Uppsala University, 751 85 Uppsala, Sweden;; 9 Department of Surgical Oncology, Medical University of Gdansk, 80-952 Gdansk, Poland;; 10 Bank of Frozen Tissues and Genetic Specimens, Department of Medical Laboratory Diagnostics, Medical University of Gdansk, 80-211 Gdansk, Poland;; 11 Surgical Oncology, Collegium Medicum, Oncology Center, Nicolaus Copernicus University, 85-796 Bydgoszcz, Poland

## Abstract

Sporadic breast cancer (SBC) is a common disease without robust means of early risk prediction in the population. We studied 282 females with SBC, focusing on copy number aberrations in cancer-free breast tissue (uninvolved margin, UM) outside the primary tumor (PT). In total, 1162 UMs (1–14 per breast) were studied. Comparative analysis between UM(s), PT(s), and blood/skin from the same patient as a control is the core of the study design. We identified 108 patients with at least one aberrant UM, representing 38.3% of cases. Gains in gene copy number were the principal type of mutations in microscopically normal breast cells, suggesting that oncogenic activation of genes via increased gene copy number is a predominant mechanism for initiation of SBC pathogenesis. The gain of *ERBB2*, with overexpression of HER2 protein, was the most common aberration in normal cells. Five additional growth factor receptor genes (*EGFR*, *FGFR1*, *IGF1R*, *LIFR*, and *NGFR*) also showed recurrent gains, and these were occasionally present in combination with the gain of *ERBB2*. All the aberrations found in the normal breast cells were previously described in cancer literature, suggesting their causative, driving role in pathogenesis of SBC. We demonstrate that analysis of normal cells from cancer patients leads to identification of signatures that may increase risk of SBC and our results could influence the choice of surgical intervention to remove all predisposing cells. Early detection of copy number gains suggesting a predisposition toward cancer development, long before detectable tumors are formed, is a key to the anticipated shift into a preventive paradigm of personalized medicine for breast cancer.

Sporadic breast cancer (SBC) affects ∼10% of women in developed countries and is a heterogeneous disease in which individual cases differ in clinical manifestation, radiologic appearance, prognosis, therapeutic possibilities, and outcome. Unlike for familial breast cancer, where mutations in a few predisposing genes in the germ line cells can be evaluated and used for disease prediction as well as choice of treatment, there is no reliable way of advanced prediction of which women in the general population are at risk for SBC later in life. The current diagnosis of SBC is made using a combination of clinical, radiological, genetic, and pathological parameters, in which molecular and histopathological evaluation of primary tumor(s) is one of the decisive determinants for the course of treatment. Survival rates for SBC vary greatly worldwide, ranging from ≥80% in North America, Sweden, and Japan to ∼60% in middle-income countries and <40% in low-income countries (Lakhani et al. 2012). Mammography screening is used for detection of tumors, but its sensitivity is limited, and it identifies a disease where primary tumors already pose a risk for mortality. The presence of multifocal tumors (i.e., multiple synchronous and ipsilateral foci) has been described in 9%–75% of SBCs, and these large discrepancies in the reported incidence are dependent on the applied definitions, mode of detection, and differences in pathological assessment (Jain et al. 2009). Multifocality in SBC is associated with increased lymph node positivity rates and worse overall outcomes compared with unifocal SBC (Tot et al. 2011).

Genetic research of SBC has been dominated by two major approaches, the first being studies of gene expression, chromosomal aberrations, and mutations in tumors, which have generated new molecular classification and confirmation of the heterogeneity of the disease (Sorlie et al. 2001; The Cancer Genome Atlas 2012; Stephens et al. 2012). The second major approach uses genome-wide association studies (GWAS) (Visscher et al. 2012), with focus on characterization of genetic variation in germline inherited genomes and identification of possible genetic predisposing factors. In the interface between these major research directions, the concept of field cancerization has evolved, in which the presence of cancer-related aberrations in various organs arises as an effect of mutation frequencies coupled with normal cell divisions and/or from exposure to carcinogens (Deng et al. 1996; Forsti et al. 2001; Heaphy et al. 2009; Bista et al. 2012; Rivenbark and Coleman 2012; Foschini et al. 2013). Recent analysis of three prostate cancer patients has shown the existence of clonal cell expansions, consistent with field effects, in morphologically normal prostate tissue (Cooper et al. 2015). Moreover, the sick-lobe concept of SBC development is a similar framework, in which early genetic aberrations are presumed to predispose specific breast lobes to cancer from early development (Tot 2005, 2014). Our current study is based on the above-mentioned encouraging results of mammary field cancerization, and we demonstrate that a comprehensive analysis of the histologically normal breast tissue (designated as uninvolved margin, UM) in SBC can lead to identification of acquired-during-lifetime, specific genetic signatures that may increase risk of SBC development.

## Results

### Wide spectrum and high frequency of genetic aberrations in uninvolved margin breast specimens

We studied 282 female SBC patients who underwent mastectomy from four oncology centers. The clinical details of the studied patients, the histopathological characteristics, and the type and number of samples studied by genetic methods are shown in Supplemental Table 1. In contrast to the common approach of studying genetic variation in tumor cells (The Cancer Genome Atlas 2012; Stephens et al. 2012), we focused on characterization of mutations in samples from macroscopically tumor-free (designated as uninvolved margin, UM) breast tissue. The definition of a UM sample is as follows: a tissue fragment noncontiguous with the tumor focus (and taken at various distances from the tumor) that, upon initial pathological macroscopic dissection (prior to fixation, paraffin embedding, and microscopic analysis), is indistinguishable from normal breast tissue. The largest distance between a primary tumor and a UM in our study was 24 cm. In total, 1162 UMs, ranging from 1 to 14 samples per patient were analyzed on Illumina arrays. For each subject, DNA from at least one control tissue was studied, which was predominantly blood, alternatively skin. We also studied primary tumor(s) (PTs), with up to three tumor foci in cases with multifocal disease.

**Figure 1.**
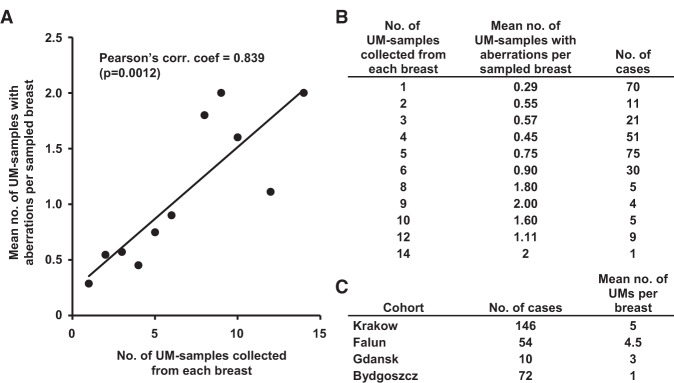
A larger number of UM samples studied per breast increases the likelihood of finding genetically aberrant UM tissue. (*A*) The number of UMs sampled per patient is positively correlated with the mean number of UMs displaying an aberrant genetic profile among all 282 studied cases of breast cancer. (*B*) Table showing the number of cases that were used for the plot in *A* and that were the basis for calculation of the correlation coefficient. (*C*) Table showing the number of cases and the mean number of UMs that were studied from each of the four participating oncology centers.

Comparative analyses between a triad of global genome profiles for blood/skin, UM, and PT is the core of the study design. Scoring of post-zygotic structural genetic variants in UMs was based on comparison of a profile for blood/skin versus a profile of UM (for each UM specimen separately) in the same patient, where changes only present in the UMs and absent in blood/skin were scored. Consequently, this study does not describe copy number alterations/polymorphisms inherited from parents via germline. We identified a total of 183 UMs with at least one aberration, and the total number of patients with at least one aberrant UM was 108, corresponding to 38.3% of all patients. The corresponding numbers by the provider institution, with at least one aberrant UM are 39.0%, 37.5%, 30%, and 29.2% for Krakow, Falun, Gdansk, and Bydgoszcz, respectively. Our data show that the number of UMs sampled per cancer-bearing breast is positively correlated with the mean number of UMs displaying an aberrant genetic profile (Fig. 1). This suggests that the uncovered post-zygotic aberrations in women with SBC represent only a part of all aberrations that might exist in the studied individuals and that mammary field cancerization is common in SBC patients.

Our primary method of detection and scoring of gene copy imbalances was genome-wide Illumina SNP chips. We used deviations in log R ratio (LRR) and B allele frequency (BAF) values as the main tool for detecting candidate aberrations because it allows the uncovering of three major types of aberrations: deletions, duplications, and copy-number-neutral loss of heterozygozity (CNNLOH; also called uniparental disomy). The additional advantage of the method is that deviation of BAF values from 0.5 for heterozygous SNP probes allows estimation of the number of cells containing a variant genotype. This platform is sensitive for detection of structural mosaicism in samples containing as few as 5% of cells with a variant genotype (Conlin et al. 2010; Razzaghian et al. 2010; Rodriguez-Santiago et al. 2010; Forsberg et al. 2012, 2013, 2014). Nevertheless, we performed 21 validation experiments on UM and PT samples from nine subjects (BK152, DH74, DM138, JU32, KK123, ME114, PI33, SE135, and ML36) by NimbleGen 720K genome-wide arrays, using standard array CGH methodology, and applying blood DNA from the same subject as a normal control sample. We also performed whole-genome sequencing of four specimens from two of these subjects. In all these analyses, we used the same DNA that was earlier examined on the Illumina platform. Supplemental Figures 1 and 2 show examples of such validation experiments from three subjects. In conclusion, there was 100% concordance between results from SNP arrays and from array CGH as well as from next-generation sequencing data in scoring of aberrations present in UMs and primary tumors.

**Figure 2.**
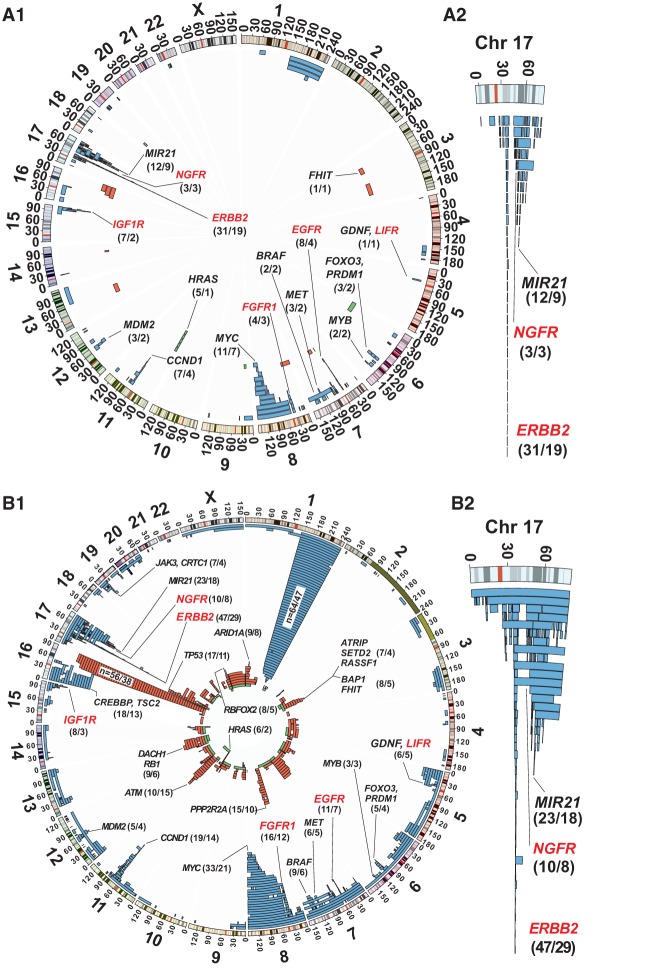
Position and frequency of post-zygotic copy number aberrations in UM samples from 282 breast cancer patients included in the study. (*A1*,*B1*) Genome-wide view of aberrations stratified by size; <105 Mb of total size and up to 1288 Mb for all size-scored aberrations, respectively. (*A2*,*B2*) An enlarged view of complex 17q amplicons, targeting *ERBB2*, *NGFR*, and *MIR21*, among other genes, which are also displayed in *A1* and *B1*. Three types of aberrations were detected using whole-genome Illumina SNP-array genotyping, such as gains (blue), deletions (red), CNNLOH/UPD (green), and are displayed using Circos plots (Krzywinski et al. 2009). Recurrent mutations including previously known cancer genes are specified by name. The numbers in parentheses after the gene names indicate the number of UM specimens and the number of cases, respectively, showing variation in each of the recurrent loci. *A1* shows the 235 structural aberrations scored in 80 UM samples collected from 50 subjects. This plot displays early aberrations, which are detected in normal UM cells, with a maximum total size of aberrations of <105 Mb. Six genes coding for cell-surface receptors showing recurrent copy number gains are highlighted in red. In *B1*, a less strict cut-off size limit was used as compared to *A1*, and 904 size-scored aberrations detected in 156 UM samples collected from 93 cases are plotted. The highly recurrent regions are all-encompassing loci previously described to be of importance in breast cancer (Table 1).

Overall, we found a wide spectrum of total aberration load for scored aberrations among the 183 UMs; the smallest total aberration load in a UM sample was 0.4 Mb and the largest involved more than half of the genome. Supplemental Table 2 shows a summary of all aberrant UMs. The size of a single scored aberration in UM samples ranged from 39 kb to 190 Mb. Figure 2, panel B1, shows the position and frequency of 904 size-determined aberrations in 156 UMs. The 27 most aberrant UMs were excluded from detailed scoring of aberrations, since they showed pronounced cancer-like profiles, heavily de-regulated on the gene copy number level, making it difficult to score all aberrations by size with a similar precision. These 27 UMs frequently contained aberrations stretching over large genomic regions (often whole chromosomes) and displayed wide differences in the number of cells affected with various copy number changes, suggesting a heterogeneity of cell clones affected by different aberrations contributing to the overall profiles. We determined that the total size of alterations in these 27 UMs was exceeding 39% of the genome (Supplemental Table 2). The results from these 27 UMs are consistent with genetic profiles of cancer cells, suggesting that upon initial pathological dissection of the resected breast, we obtained samples from additional focus/foci of breast cancer. In summary, we could detect genetic aberrations in any of the UM samples in nearly 40% of patients, which suggests that the process of mammary field cancerization is common. We have also examined on Illumina arrays a series of 48 samples of normal breast tissue derived from reduction mammoplasty specimens of women without any suspicion or diagnosis of breast cancer. The profiles of all these samples were normal, without any indications of recurrent copy number changes that were observed in UMs from women with breast cancer (details not shown).

**Table 1.**
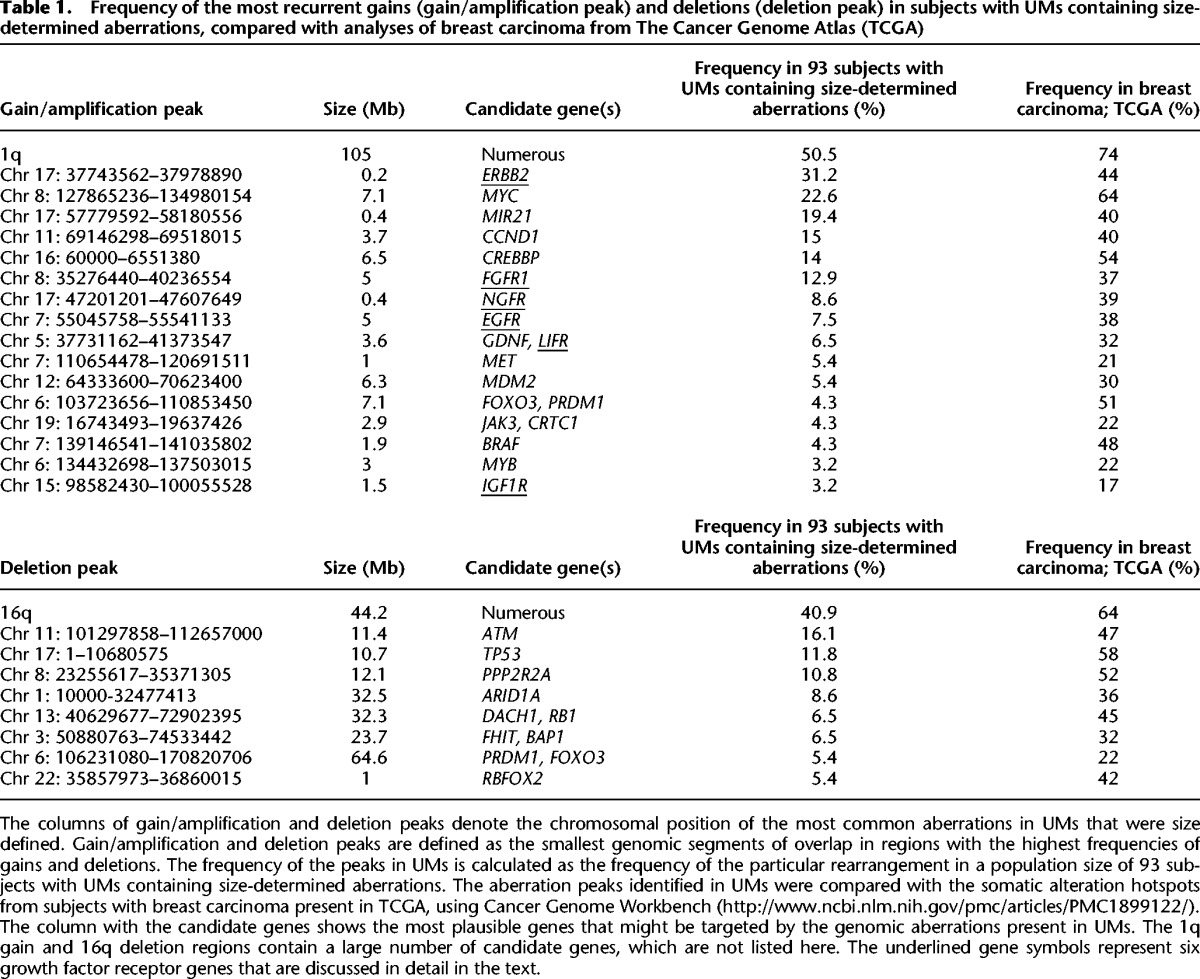
Frequency of the most recurrent gains (gain/amplification peak) and deletions (deletion peak) in subjects with UMs containing size-determined aberrations, compared with analyses of breast carcinoma from The Cancer Genome Atlas (TCGA)

### Correlation between the total load of aberrations in UMs and histological findings

We visualized the aberrations scored in UMs using size stratification according to the total load of aberrant genomes. The size-defined aberrations in 156 UMs are shown in panels A1 and B1 in Figure 2 using two thresholds: ≤105.6 Mb of total aberration load and up to 1288 Mb, respectively. The rationale for this stratification is based on recent studies describing clonal expansions of normal blood cells in aging humans, which show that genetic aberrations in normal cells can be of considerable size (Forsberg et al. 2012, 2014; Jacobs et al. 2012; Laurie et al. 2012). The largest aberration observed so far in the peripheral blood of healthy subjects was a mosaic gain of Chromosome 3, i.e., an alteration with a total size of ∼200 Mb (Forsberg et al. 2014). Our rationale here was to establish a threshold of aberration load compatible with normal breast tissue histology and attempt identification of specific genes involved in the generation of aberrations in normal epithelial cells. We used a combination of complementary approaches toward this goal: (1) genetic analysis of tissue from laser-microdissection (LMD); (2) histological analysis of UMs with low aberration load; (3) similar study of UMs with high aberration load; and (4) large-format histopathology sections with focus on UMs with various aberration loads. These results are presented in the paragraphs below.

We used LMD followed by Illumina genotyping, allowing genetic analysis of histologically well-defined samples and permitting a comparison of results from bulk DNA from the same UM tissue specimen. The set-up for LMD experiments was dissection of >200,000 cells and isolation of DNA in the range of 1-µg quantity, facilitating SNP-array genotyping without introducing an additional step of genome amplification, which could be combined with a risk of artifacts. We initially tested SNP genotyping of LMD-derived DNA in three subjects and started with tissue derived from mammoplasty of a woman without suspicion of SBC. Supplemental Figure 3 shows the histological and genetic analyses of this case. The genotyping was of high quality (SNP call rate > 98% and LogRdev value < 0.2), and the genomic profile derived from the LMD experiment was normal and identical to the profile derived from bulk DNA from the same specimen. We further tested the LMD methodology using two SBC patients, where histological analysis of UMs indicated the presence of low-grade carcinoma in situ (sample 100AW-VB) and a mixture carcinoma in situ with invasive ductal carcinoma (sample 085AS-VB). The genetic analysis of bulk DNA from these UMs showed many genetic aberrations, and the comparative analyses are shown in Supplemental Figures 4 and 5. For example, bulk DNA of UM from case 085AS contains numerous aberrations, but these occur in a relatively low percentage of studied cells. LMD-derived DNA shows an enrichment of cells with abnormal copy number profiles. Case 100AW is also illustrative in terms of enrichment of cells with changes on Chromosomes 8 and 16, which are reaching 100% in LMD-derived DNA. However, three aberrations on Chromosomes 1, 11, and 19 are not confirmed in LMD-derived DNA, which suggests that the bulk DNA from this sample is a heterogeneous mixture of several cell clones with distinct genotypes. It is also noteworthy that the bulk DNA from UM sample 100AW contains a larger number of copy number changes, when compared with PT.

**Figure 3.**
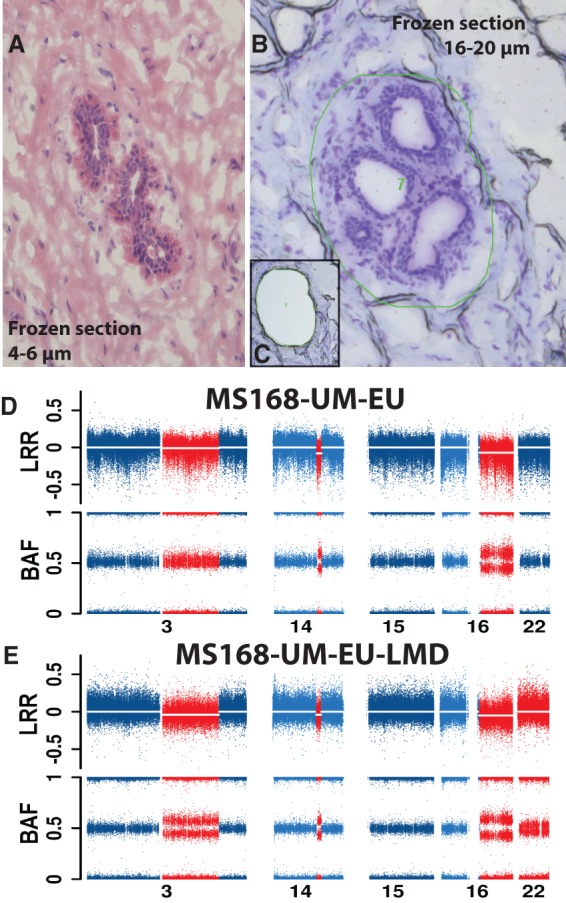
Laser-microdissection (LMD) validation of three deletions on Chromosome 3, 14, and 16 in normal cells from sample MS168-UM-EU. (*A*) A representative image of normal breast parenchyma (hematoxylin and eosin staining) in thin frozen section from specimen MS168-UM-EU, with a normal duct. (*B,C*) Images before and after the normal structures have been dissected by laser and collected. The thick frozen sections (16–20 µm) in *B* and *C* have been stained with cresyl violet. The green irregular circle in *B* shows the area marked for dissection by laser. (*D*,*E*) Genetic copy number profiles of chromosomes with aberrations (in red) and without (in blue) from SNP arrays. The profile in *D* has been produced using the bulk DNA derived from all cells in sample MS168-UM-EU, while the profile in *E* is derived from DNA isolated from microdissected cells. Sample MS168-UM-EU shows deletions present in ∼5%–15% of cells, as indicated by the BAF values deviating from the value of 0.5. The corresponding number of cells affected by deletions in sample MS168-UM-EU-LMD is higher, suggesting an enrichment of cells with aberrations. The combined load of deletions on Chromosomes 3, 14, and 16 in the sample MS168-UM-EU is 92.8 Mb. Interestingly, the microdissected sample MS168-UM-EU-LMD contains also a low proportion of cells (∼5%–10%) with a copy number neutral loss of heterozygozity (CNNLOH) of whole Chromosome 22, which was not detectable in the bulk DNA derived from all cells in sample MS168-UM-EU.

Two additional patients with a small aberration load in UMs and normal histology were validated by LMD (EG163-VB2 and 131SD-UM-IL) involving a 7.8-Mb gain (*ERBB2*) and a 13.7-Mb deletion (*DMTF1* tumor suppressor), respectively (Supplemental Figs. 6, 7). The LMD validations were further extended to four cases with a larger total load of aberrations in histologically normal UMs, ranging from 92.8 to 105.6 Mb in several samples (Fig. 3; Supplemental Figs. 8–10). Detailed comparisons of results between bulk UM-DNA and LMD-derived DNA from cases MS168-UM-EU and EW155-UM-IL2 show that dissected cells contain additional alterations (on Chromosomes 22 and 9, respectively) that were not detectable in the bulk UM-DNA. A plausible explanation of this result is that these UMs contain additional changes in cell clones that were not analyzed in the bulk UM-DNA. This further reinforces the notion that genetic heterogeneity of various cell clones within histologically normal breast parenchyma from breast cancer patients is underestimated.

We further performed detailed histologic and genetic analysis of UMs in an additional 18 patients with a wide range of total aberration load. Six of these are shown in Supplemental Figures 11–16, where histologically normal ducts and terminal ductal lobular units contained various genetic changes ranging from 1.8 to 173.1 Mb in total size. Supplemental Figure 17 shows breast tissue in a UM from case 063JB. The total size of aberrations in the 063JB-VB sample was 143.8 Mb, and it contained a mixture of areas with low-grade carcinoma in in situ cells and normal ducts. The next case in ascending order of total size of aberrant genome was 100AW-UM-IU, containing 193 Mb aberrations and a ductal carcinoma in situ (DCIS) (not shown). The additional 10 cases analyzed in the same manner had even higher total aberration loads, and all contained either DCIS or a mixture of DCIS and invasive carcinoma or exclusively invasive carcinoma cells. These are 049ASZ-VB, 306 Mb, DCIS/invasive ductal carcinoma (IDC); 095ESZ-UM-EU, 317 Mb, DCIS; 017KM-VB, 446 Mb, IDC; KK151-UM-EU2, 486 Mb, DCIS/IDC; 100AW-VB, 532 Mb, DCIS (Supplemental Fig. 4); 085AS-VB, 730 Mb, DCIS/IDC (Supplemental Fig. 5); 081BS-UM-EU, 823 Mb, DCIS; 086AFT-VB, 1287 Mb, DCIS; 141BB-VB2, >39% of the genome, invasive lobular cancer; and JP149-UM-EU2, >39% of the genome, IDC.

We also examined all patients of the Falun clinic using the large-format histopathology sections. This powerful platform of large paraffin-embedded contiguous breast tissue slices (up to 10 × 24 cm) allows analysis of histology of tissue surrounding UMs and exact localization of the site of UM sampling of fresh tissue for genetic analysis, in relation to the position of PT sample. We especially focused this analysis on 45 UMs showing aberrant genetic profiles. In all but six UMs (indicated by a zero in the column showing the exact distance from PT(s); Supplemental Table 2), these were free from tumor/atypical cells at the UM-sampling site. The sample AL002_UM1 contained four aberrations on three chromosomes with a total aberration load of 107.6 Mb. This important UM represents the case with the smallest aberration load in tissue with detectable cancer/atypical cells in our study.

In summary, the total aberration load in UMs and their specific genomic locations seems to influence and correlate with the histological findings. The largest total size of aberrant genome (173.1 Mb) in tissue with normal morphology was KM159-UM-IU1 (Supplemental Fig. 16) and the UM sample AL002_UM1 represents the case of smallest aberration load (107.6 Mb) in tissue with detectable cancer cells. Consequently, an aberration load below ∼105 Mb in UM(s) could be considered a signature of SBC predisposition that is acquired during lifetime. The seven most frequent candidate genes that are located within altered regions in UMs with low aberration load (<105 Mb) were affecting the following genes: *ERBB2*, *MIR21*, *MYC*, *VMP1*, *EGFR*, *IGF1R*, and *CCND1* (Fig. 2, panel A1; Supplemental Table 2). It should be stressed that gains were the principal type of alteration in UMs with low aberration load; these represented 92.3% of all aberrations in this category. The corresponding numbers for deletions and CNNLOH are 4.2% and 3.4%. This result suggests that oncogenic activation (up-regulation) of genes via increased copy number might be a predominant mechanism for initiation of the SBC disease process. It is also noteworthy that UMs with aberration load <105 Mb already display two of the most common larger-scale chromosomal changes found in all UMs and PTs in this study, i.e., gain of 1q and deletion of 16q (Table 1; Fig. 2, panel A1; Supplemental Table 2). However, as opposed to the picture very frequently observed in breast carcinomas, 1q-gain and 16q-deletion were never observed together in the same UM sample with normal histology. This is compatible with an additive effect of these two rearrangements in transforming normal breast epithelial cells into tumor cells.

### Propagation of genetic aberrations from UMs into PTs

We examined whether the genomic alterations in UMs affecting specific regions/genes were also present in PTs from the same patients and found this to be a rule with only two exceptions. One exception is shown in Supplemental Figure 7; case 131SD-UM-IL. This 13.7-Mb deletion, targeting the *DMTF1* tumor suppressor gene (Inoue et al. 2007), which was the only aberration in this UM, was not propagated into PT. The other exception was the case 100AW-VB (Supplemental Fig. 4), where the UM showed eight copy number changes, but only four of these were propagated into the PT sample. It is noteworthy that the PT of this case contained a lower number and had a lower total aberration load, when compared with the UM sample (100AW-VB). These two cases suggest that UMs may contain genetic changes causing clonal expansions of affected cells. However, these aberrations may not always be causative in the development of a primary tumor. This is reminiscent of our results and those of others showing that clonal cell expansions in normal blood are common and do not always lead to development of a clinical phenotype in subjects carrying such clones (Forsberg et al. 2012, 2013, 2014; Jacobs et al. 2012; Laurie et al. 2012; Holstege et al. 2014; Score et al. 2015). Furthermore, as shown in multiple figures, the aberrations detected in both UMs and PTs were present in PT samples in a considerably higher percentage of cells and were often accompanied by many additional aberrations that were present in PTs only. In summary, the above results allow interpretation of the UM-associated events as precursors in a lineage leading to the primary tumors.

### The aberrations in UMs mirror copy number alterations previously described in breast carcinomas

In order to identify the important genes affected by copy number aberrations in all aberrant UMs and to compare these with the aberration hotspots already described in the literature of breast cancer, we have characterized peaks of copy number gain and loss (Table 1). Gain/amplification- and deletion peaks were defined as the smallest overlap in the segment most often affected by gains and deletions. The frequency of peaks in UMs was calculated as the occurrence of a particular rearrangement in the population size of 93 subjects displaying a size-defined aberration(s). The gain/amplification peaks were, in a majority of cases, limited in size (from several hundred kilobases to a few megabases) and consequently contained a limited number of candidate genes. These are: *ERBB2, MYC, MIR21, CCND1, CREBBP, FGFR1, NGFR, EGFR, GDNF/LIFR, MET, MDM2, FOXO3/PRDM1, JAK3/CRTC1, BRAF, MYB*, and *IGF1R.* The aberration peaks for deletions were considerably broader, spanning 10 to 65 Mb in size, with the exception of a deleted segment of ∼1 Mb on 22q, containing, among others, the *RBFOX2* gene. In other cases (for instance, the frequent 16q-deletion), the aberrant regions were too large and contained too many genes to identify specific candidates.

We compared our observations in UMs to the somatic alteration hotspots in breast carcinoma from The Cancer Genome Atlas (TCGA) (The Cancer Genome Atlas 2012) using The Cancer Genome Workbench (Zhang et al. 2007). As expected, the landscape of aberrations observed in UMs reflected the hotspots described in tumors, although with lower frequencies (Table 1). Overall, we describe a strong concordance between the two data sets, with 1q-gain and 16q-deletion being the most common copy number changes (Table 1). Thus, the recurrent aberrations identified in UMs have all previously been described in breast tumors and other cancers, suggesting their causative, driving role in the disease process. In a few instances, we observed a considerably higher frequency of gains in TCGA compared to UMs in our data set. For instance, a region of Chr 6: 103723656–110853450, containing *FOXO3* and *PRDM1*, displayed a frequency of 51% and 4.3% in TCGA and UMs, respectively. Similarly, Chr 7: 139146541–141035802, with *BRAF*, was found at a frequency of 48% in TCGA and 4.3% in UMs. Furthermore, we scored only a few 20q-gains in UMs (Fig. 2). TCGA data set shows, however, that gain/amplification of 20q is observed in 42%–47% of cases, suggesting also that this event is likely to occur at later stages of breast carcinogenesis. In summary, these results may suggest that such alterations are usually not the early cancer-predisposing events but rather later changes acquired by already transformed cells.

### Aberration load of UMs and their distances from PT(s)

**Figure 4.**
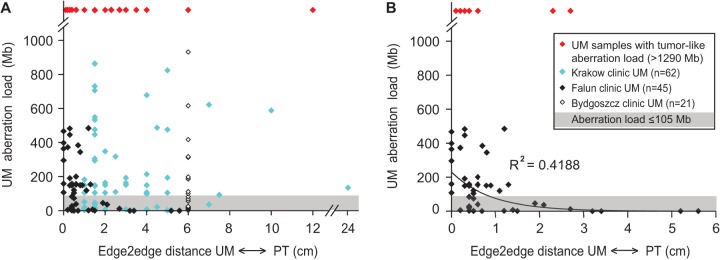
The total aberration load of UM samples in relation to the distances between UMs and PTs. The “edge2edge” distance was measured as the shortest distance between the borders of the PT and UM samples. For patients with multifocal disease, the distance was measured to the closest primary tumor. In *A*, combined data from three clinics (Krakow, Falun, and Bydgoszcz) are shown. (*B*) Falun cases only. The shaded area in both plots illustrates the 105-Mb threshold as defined by our comparative genetic and histological analysis that is described in the text. In our material, no UM samples with an aberration load below the 105-Mb threshold showed any atypical/cancer-like features upon microscopic inspection. Red diamonds highlight UMs in which the total aberration load (i.e., >1288 Mb) was indicative of tumor content in these samples, as explained in the text. The symbols for samples derived from each of the clinics are explained in the box in *B*. The distances for the Falun cases were measured in a microscope using a large-scale histology format, allowing high precision of measurements, i.e., below 1 mm accuracy. The distances for the other two clinics were measured with a ruler upon dissection of the breast by a pathologist and are less precise. In six instances of UM samples from the Falun clinic, the microscopic investigation of large-format histology preparations resulted in detection of tumor/atypical cells in the area where UM samples were taken, and these UMs are plotted at zero distance from the primary tumor. The trend line was introduced for Falun cases with an *R*^2^ value of the correlation coefficient. The UM samples from the Bydgoszcz clinic were collected at a 4- to 8-cm distance from PTs and we used the average distance in this plot, as reflected by the cluster of measurements at the 6-cm distance in *A*. The plotted data can be found in Supplemental Table 2.

We studied the distances between PTs and genetically aberrant UMs, also considering the observed aberration load of UMs. The distance between the PT and UM was measured as the shortest “edge2edge” distance between the borders of PT and UM samples. For patients with multifocal disease, the distance was measured to the closest PT. Our rationale and assumptions were twofold: (1) If the UMs with low aberration load (<105 Mb) were involved in acquired predisposition to develop SBC, then these samples (representing histologically normal tissue) would be spread at various distances from PTs and would also be located at large distances from tumors; and (2) if the process of contamination from infiltrating nearby-located tumor(s) was responsible for the aberrant profiles seen in UMs, then the heavily aberrant tumor-like UMs would concentrate very close to PTs. Figure 4 displays the results from our analysis, providing a complex picture. The first assumption is largely supported by the data. The UMs with low aberration load (<105 Mb) (shaded fields in Fig. 4) are located at highly variable distances from primary tumor. The second assumption, however, is not clearly supported. While there are many UMs with high aberration load among those located in the immediate vicinity (<1 cm) of PTs, these tumor-like UMs are also spread at considerable distances, the largest being 12 cm. One plausible explanation for the tumor-like UMs that are located at very large distances from histopathologically diagnosed PTs is the underestimated importance of multifocality in the pathogenesis of SBC.

### Low copy number gain of the *ERBB2* gene and HER2 protein expression occurs in microscopically normal epithelial and mesenchymal cells from breasts of SBC patients

Low copy number gain of the *ERBB2* gene was the most common event among UMs with <105 Mb aberration load (Fig. 2, panels A1,A2) and the third most common change among all studied UMs (Table 1; Fig. 2, panels B1,B2). It has recently been suggested that the gain of *ERBB2* in normal cells in the vicinity but outside the focus of primary tumor might represent an event related to infiltration of cancer cells into the normal parenchyma (Sadanandam et al. 2012). We therefore studied whether this aberration is present in microscopically normal epithelial cells, taking advantage of the large-format histopathology sections from Falun. These large, paraffin-embedded contiguous breast tissue slices allowed exact localization of the site of sampling of fresh tissue cores for genetic analysis. New tissue samples were taken from the immediate vicinity of these biopsy sites for morphological *ERBB2*/HER2 assessment. We applied this strategy in the combined analysis of *ERBB2* expression and copy number analysis using the HER2 tricolor Dual ISH DNA Probe Cocktail Assay, allowing visualization of expression of the HER2 protein as well as the copy number variation of *ERBB2* and the centromere of Chromosome 17 (Nitta et al. 2012). Eleven cases from the Falun cohort were selected for this analysis, based on the results of *ERBB2* analysis in UM samples from the Illumina platform. PTs from all these subjects were characterized as either HER2-positive or Luminal B HER2-positive.

**Figure 5.**
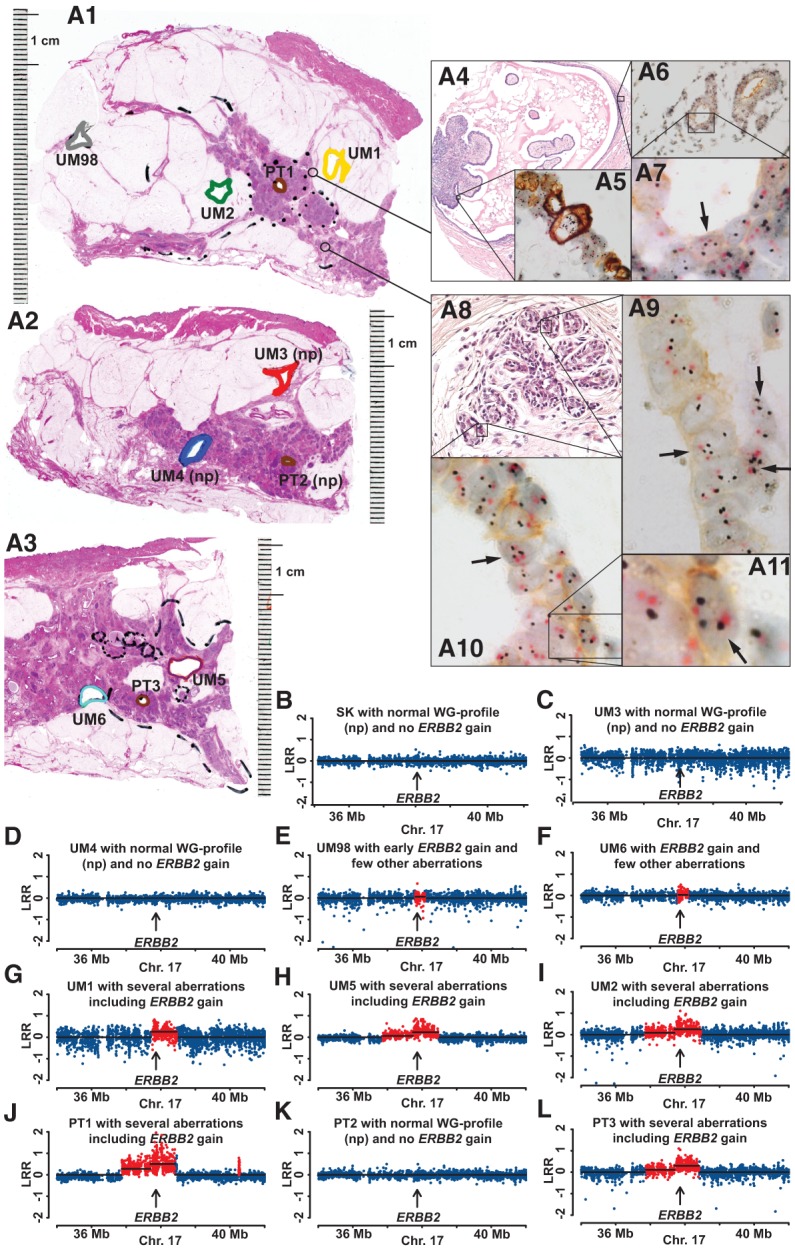
Comprehensive study of pathology and genetics for case MN036, showing increased copy number and expression of the *ERBB2* gene in normal epithelial cells. (*A1*,*A2*,*A3*) Three large-format histology slides taken at different levels of the mastectomy specimen stained with hematoxylin and eosin, with diagnosis of multifocal invasive ductal carcinoma (Luminal B, HER2+). Areas of tissue samples taken for DNA extraction, prior to formalin fixation of the tissue, are marked with colored thick lines. Positions of three primary tumors 1, 2, and 3 (PT1, PT2, and PT3) are shown in brown. In *A1*, UM1, UM2, and UM98 are labeled in yellow, green, and gray, respectively. In *A2*, UM3 and UM4 are labeled in red and blue, respectively. In *A3*, UM5 and UM6 are labeled in purple and light blue, respectively. (np) Normal genetic profiles (see also below, *B*–*L*). Two cores from paraffin-embedded tissue (thin-lined black circles) from *A*1 were taken for separate analysis using the HER2 tricolor Dual ISH DNA Probe Cocktail Assay (Roche) and the results are shown in *A4*–*A11*. (*A4*) A papillary structure lined partly by cancer cells and partly by histologically normal epithelium. High-magnification image in *A5* shows tumor cells with very strong overexpression of HER2 protein containing up to 20 copies of *ERBB2* (black dots). (*A6*,*A7*) Histological images of normal breast tissue. Black arrows in *A7*, *A9*–*A11* point to single nuclei of normal epithelial cells containing more than two copies of *ERBB2* (black dots). The centromere of Chromosome 17 is stained in red. A weak but clearly discernible immunohistochemical staining of HER2 protein is visible in the cell membrane of normal epithelial cells upon high magnification. (*B*–*L*) A segment of Chromosome 17 containing *ERBB2* in 11 samples from Illumina global genome analysis. Skin (SK, normal control tissue), UM3, UM4, and PT2 show no evidence of gain of *ERBB2*. The remaining seven samples were scored as containing increased copy numbers (red dots) for *ERBB2*. Note that sample UM98, located at a distance of >4 cm from the PT1 sample, also shows evidence for cells containing an increased number of copies of *ERBB2*. The total size of aberrations in UM samples is as follows: UM6, 0.4 Mb; UM98, 0.4 Mb; UM1, 0.8 Mb; UM2, 27.7 Mb; UM5, 36.5 Mb.

**Figure 6.**
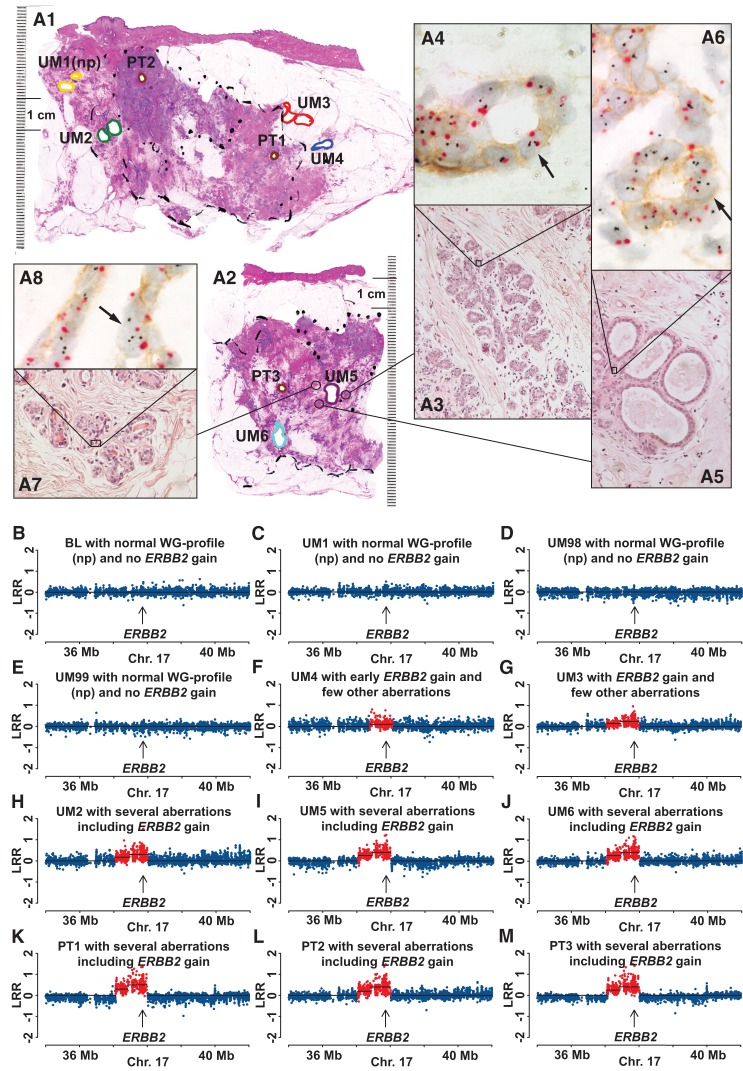
Multimodal examination of pathology, gene copy number, and gene expression for case AW020, with evidence of increased copy number and expression of *ERBB2* in normal epithelial cells. (*A1*,*A2*) Two section levels of large-format histology slides of breast tissue stained with hematoxylin and eosin, with diagnosis of multifocal invasive ductal carcinoma (Luminal B, HER2+). Areas of tissue samples taken for DNA extraction, prior to formalin fixation of the tissue, are marked with colored thick lines. Positions of three primary tumors 1, 2, and 3 (PT1, PT2, and PT3) are shown in brown. In *A1*, UM1, UM2, UM3, and UM4 are labeled in yellow, green, red, and blue, respectively. In *A2*, UM5 and UM6 are labeled in purple and light blue, respectively. (np) Normal genetic profiles (see also below, *B*–*M*). Three cores from paraffin-embedded tissue (thin-lined black circles) surrounding sample UM5 were taken for separate analysis using the HER2 tricolor Dual ISH DNA Probe Cocktail Assay (Roche) and the results are shown in *A3*–*A8*. (*A3*,*A5*,*A7*) Histological images of normal breast tissue with cross-sections through terminal ductal lobular units (TDLUs). Black arrows in *A4*, *A6*, and *A8* point to single nuclei of normal epithelial cells containing more than two copies of *ERBB2* (black dots). The centromere of Chromosome 17 is stained in red. Note a weak but clearly discernible immunohistochemical staining of HER2 protein in the cell membrane of normal epithelial cells upon high magnification. (*B*–*M*) A segment of Chromosome 17 containing *ERBB2* in 12 samples from Illumina global genome analysis. Blood (BL, normal control tissue), UM1, UM98, and UM99 samples have normal profiles (np) with no gain of *ERBB2*. The remaining eight samples were scored as containing an increased copy number (red dots) for *ERBB2*. The samples UM98 and UM99 are taken from parts of breast tissue as far away as possible from the segment (lobe) affected by breast cancer and are not visualized in *A1* and *A2*. The total size of aberrations in UM samples is as follows: UM4, 0.7 Mb; UM3, 7.4 Mb; UM2, 191 Mb; UM5, >39% of the genome; UM6, >39% of the genome.

**Figure 7.**
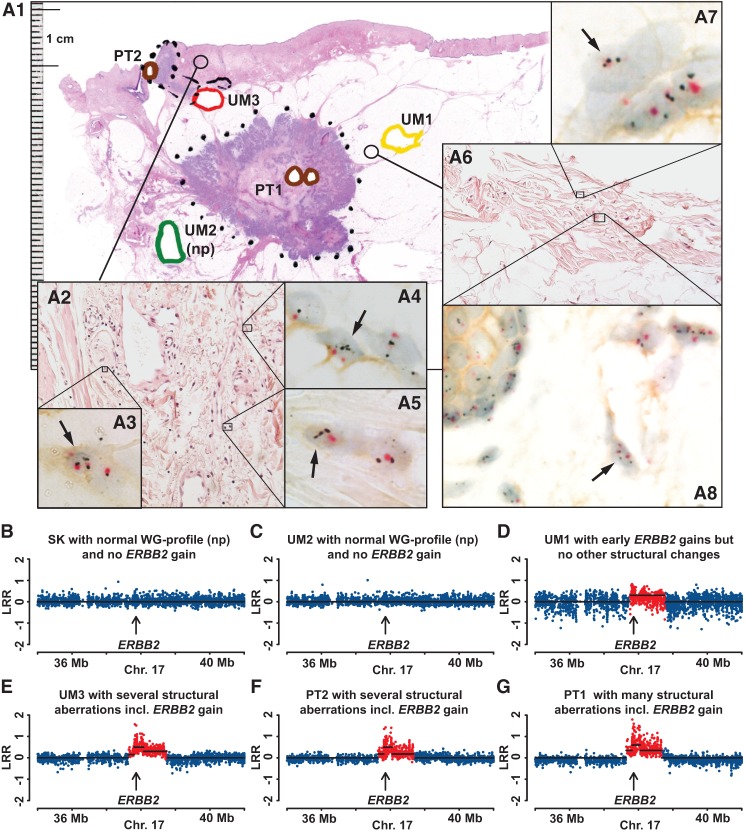
Comprehensive analysis of pathology, gene copy number, and gene expression for case MA018, showing evidence for increased copy number and expression of *ERBB2* in normal epithelial as well as in normal mesenchymal cells. (*A1*) A large-format histology slide of breast tissue stained with hematoxylin and eosin, with diagnosis of multifocal invasive ductal carcinoma (HER2+, non-luminal). Areas of tissue samples taken for DNA extraction, prior to formalin fixation of the tissue, are marked with colored thick lines. Positions of two primary tumors 1 and 2 (PT1 and PT2) are shown in brown. UM1, UM2, and UM3 are labeled in yellow, green, and red, respectively. (np) Normal genetic profiles (see also below, *B*–*G*). Two cores from paraffin-embedded breast tissue (thin-lined black circles) surrounding samples UM1 and UM3 were taken for separate analysis using the HER2 tricolor Dual ISH DNA Probe Cocktail Assay (Roche) and the results are shown in *A2*–*A8*. Black arrows point to single nuclei of normal mesenchymal stromal cells and epithelial cells containing more than two copies of *ERBB2* (black dots). The centromere of Chromosome 17 is stained in red. Note a weak but clearly discernible immunohistochemical staining of HER2 protein in the cell membrane of normal mesenchymal and epithelial cells upon high magnification. (*B*–*G*) A segment of Chromosome 17 containing *ERBB2* from six samples from Illumina global genome analysis. Skin (SK, normal control tissue) and UM2 samples have normal profiles (np) with no gain of *ERBB2*. The remaining four samples were scored as containing an increased copy number (red dots) for *ERBB2*. The total size of aberrations in UM samples is as follows: UM98, 0.7 Mb (not shown in this figure); UM1, 0.9 Mb; UM3, >39% of the genome.

In all 11 studied cases, the presence of more than two copies of *ERBB2* and overexpression of the HER2 protein could be detected in a fraction of the normal epithelial cells. The frequencies of epithelial cells having more than two copies of *ERBB2* varied from <1%–10% of all cells that were scored under high-resolution microscopy. Figures 5–7 and Supplemental Figure 18 show details of these analyses in four subjects. These figures show images of nuclei from single epithelial cells with three or more copies of *ERBB2* as well as weak but clearly discernible membranous staining of HER2 protein. This validates the results from the analysis of fresh-frozen tissue on Illumina copy number profiling. It also suggests that the deviations of *ERBB2* are among the earliest post-zygotic aberrations predisposing to and initiating the disease in these cases. Unexpectedly, we noticed that the increased copy number of *ERBB2* was not restricted to epithelial cells but also occurred in mesenchymal cells, as shown in case MA018 (Fig. 7, panels A3–A5, A8). The *ERBB2* gain was more pronounced among mesenchymal cells (8% of counted stromal cells) compared to epithelial cells (∼5% of counted epithelial cells). We analyzed this issue in all 11 cases and could determine that low copy number gain of *ERBB2* in mesenchymal cells was not restricted to case MA018 but could be observed in all 11 cases, with a variable percentage of affected cells. Case MH016 showed a similar number (∼5%) for both epithelial and mesenchymal cells in the increased copy number of *ERBB2.* In all the remaining cases, the epithelial cells showed a higher number of cells with *ERBB2* gain, compared to mesenchymal cells. Currently, the functional importance of the presence of more than two copies of *ERBB2* and overexpression of the HER2 protein in normal mesenchymal cells for the biology of breast is not clear, but this issue should be studied further. In summary, the above shows that the early predisposing genetic signatures are present in normal breast parenchyma as an expression of field cancerization and are not likely to be derived from migrating tumor cells.

### Five additional cell membrane-bound receptors (LIFR, EGFR, FGFR1, IGF1R, and NGFR) show gains in normal breast cells

The gain of the *ERBB2* locus was the most common event in 80 UMs and 50 patients with total aberration load < 105 Mb; 38.7% and 44%, respectively (Fig. 2, panels A1,A2). However, we observed other recurrent gains targeting five additional cell membrane-bound receptors; *LIFR*, *EGFR*, *FGFR1, IGF1R*, and *NGFR* (Supplemental Table 3), suggesting that they are likely overexpressed (Santarius et al. 2010). Supplemental Figures 12 and 13 show examples of two cases with gains restricted in genomic size affecting the *IGFR1* gene in normal breast cells (GC147-UM-IU1 and MW158-UM-EU1). In both cases, the *IGF1R* gains were present in multiple UMs and in a very high percentage of cells in PTs, which indicates propagation of aberrant clones. In case GC147 of Luminal A cancer, three UMs had *IGF1R* gain (C147-UM-IU2, GC147-UM-IU1, and GC147-VB1). The latter two samples also show a coexisting gain of another receptor gene (*FGFR1*) (see Supplemental Fig. 19, which describes the sample collection scheme for the Krakow cohort, and Supplemental Table 2). Case MW158 with Luminal B (HER2 negative) SBC is also illustrative because six UMs had aberrant profiles and four of these showed *FGFR1* gains (MW158-VB2, MW158-UM-EU1, MW158-VB1, MW158-UM-IU1) (Supplemental Table 2). Two of the six genetically aberrant UMs were sampled from different quadrants of the breast, compared with the one that was affected by the histopathologically diagnosed tumor. This suggests that multiple breast lobes are likely affected by genetic alterations (Supplemental Fig. 13), further emphasizing the issue that large parts of breast parenchyma can contain genetic aberrations, some of which might predispose to tumor development. A similar scenario of low copy number gains in UMs and PTs was also observed for other receptor genes, such as *FGFR1* and *EGFR*. Supplemental Figure 11 shows the case 053KS-VB, displaying *EGFR* gain in UM and PT samples. This gain is present in low (5%–15%) and high proportion (75%–90%) of cells in UM and PT samples, respectively. Supplemental Figure 16 displays case KM159, where gain of *FGFR1* was detected in UM and PT samples, with a similar relationship regarding the number of cells with *FGFR1* gain.

We further analyzed the separate and cumulative frequency of these gene activations (via gains) encompassing the six receptors discussed above (Supplemental Table 3). This was assessed in two separate categories of total aberration load: (1) <105 Mb (80 UMs from 50 patients); and (2) all size-defined aberrations scored in 156 aberrant UMs from 93 subjects. A gain of one or more of these receptor genes was present in 60% of studied patients within the category of <105 Mb total UM aberration load (Supplemental Table 3B). The most common combination of gains was *ERBB2* and *NGFR* (10%), as well as *ERBB2* together with *FGFR1* (8%) (Supplemental Table 3C).

### Correlation between genetic aberrations in UMs and molecular subtypes of primary tumors

We also studied the pattern of all size-defined genetic aberrations uncovered in UMs in relation to the molecular phenotype of primary tumors, as classified by the four oncology centers. This phenotypic classification of tumors was performed according to the St. Gallen 2011 standard (Goldhirsch et al. 2011). In a similar way as shown in Figure 2, we visualized three types of aberrations detected using SNP-array genotyping. The stratification was performed for the four main molecular phenotypes: Luminal A; HER2(+); Luminal B; and Triple Negative (Basal-like). We further subdivided the Luminal B into Luminal B-HER2(+) and Luminal B-HER2(−), generating six plots shown in Supplemental Figure 20. This analysis was informative with regard to distinguishing between at least two major types. Luminal A subtype was characterized by a high frequency of UMs with 1q-gains and 16q-deletions and low abundance of other events. Luminal B had a high frequency of gains on Chromosome 8 targeting the *MYC* proto-oncogene and the *CCND1* and *ERBB2* genes, in addition to a high frequency of 1q-gains and 16q-deletions. This picture is even more pronounced in cases with the Luminal B-HER2(−) phenotype, except for *ERBB2* gain. These results suggest that the genetic abnormalities in normal breast tissue of SBC patients occur in all molecular tumor subtypes (although in varying frequencies) and might be associated with the molecular phenotype of the tumors. Extension of our analyses using a larger number of SBCs could therefore have a future impact on the molecular diagnosis that the breast cancer patient eventually would receive.

## Discussion

Our results demonstrate that acquired-during-lifetime structural genetic aberrations affecting well-known cancer genes are common in cancer-free breast tissue (UM) sampled at various distances from PT in SBC patients. We collected up to 14 UMs per cancer-bearing breast from 282 patients, and 108 of these showed at least one aberrant UM, corresponding to nearly 40% of all patients. There was a strong correlation between the numbers of UMs studied per patient and the number of identified UMs with aberrations (Fig. 1). Although we typically studied multiple UMs per breast from a single patient, only a minute fraction of the epithelial cells present in each cancer-bearing breast was studied. It is therefore likely that the disease process is frequently affecting considerable portions of cancer-affected breasts and that the above numbers are an underestimate. These results support the role of field cancerization in the development of SBC (Deng et al. 1996; Forsti et al. 2001; Heaphy et al. 2009; Rennstam et al. 2010; Bista et al. 2012; Rivenbark and Coleman 2012; Foschini et al. 2013; Ronowicz et al. 2015) and are in agreement with the sick-lobe concept for the origin of SBC (Tot 2005, 2014). Our analysis represents a snapshot picture of a progressive process that is likely going on for many years, if not decades, eventually producing clinically and/or radiologically detectable PT(s). This assumption of a long latency time of SCB studied here should be viewed in the perspective of the estimated latency time for radiation-induced breast cancer, that is, 10–30 yr (Thomas et al. 1994; Goss and Sierra 1998; Olsson et al. 2003).

In 23 patients (8.1%), at least one UM displayed copy number alterations involving >39% of the genome, suggesting that these samples are composed of genetically highly abnormal, tumor-like cells, and all validation experiments of such UMs using histopathology support this conclusion. Thus, these UMs represent additional tumor foci that were not taken into account when patient diagnosis was made. Furthermore, a large number of microscopic validations were performed on additional UMs that had an aberration load between 105 and 1288 Mb (3.2% and 39% of the genome, respectively). These results suggest that normal-appearing tissue, noncontiguous with the tumor and containing an aberration load of >3.2% of the genome, often contains atypical/carcinoma in situ/invasive tumor cells. This should be discussed in the context of the multifocality of SBC and local therapeutic failure seen in a considerable proportion of SBCs (Huston and Simmons 2005). Multiple synchronous and ipsilateral cancer foci have been described in 9%–75% of SBC patients (Jain et al. 2009), and multifocality in SBC is associated with increased lymph node positivity rates and worse overall outcomes compared with unifocal SBC (Tot et al. 2011). Another unexpected and puzzling result from our analysis is that microscopically normal mesenchymal cells, in addition to epithelial cells, contained *ERBB2* gains. The biological significance of this finding is unknown and deserves further study.

An important question in mammary field cancerization is the mechanism of development and origin of cells carrying genetic aberrations that are present in breast tissue with normal histology. The issue of a possible contamination of UMs with cancer cells due to an infiltrative process of tumor spread, occurring in three dimensions, is therefore crucial. For example, it has been proposed that aberrations found in normal breast tissue could have originated from migrating tumor cells (Sadanandam et al. 2012), and we have therefore examined this possibility by a combination of several complementary approaches. First, we established by genetic analyses of cells harvested using laser-microdissection from UMs that the detected mosaic structural genetic aberrations are indeed derived from microscopically normal epithelial cells (Fig. 3; Supplemental Figs. 4–10). Second, we used large-format histology for parallel analyses of copy number status of the *ERBB2* gene located on Chromosome 17, independent assessment of copy number status of Chromosome 17, as well as expression levels of the *ERBB2*-encoded HER2 protein in 11 cases (Figs. 5–7; Supplemental Fig. 18). As illustrated in these figures, microscopically normal cells can contain two to five copies of *ERBB2*, while tumor cells from the same paraffin-embedded tissue section could contain up to 30 *ERRB2* gene copies. It is unlikely that tumor cells could have migrated to another location, reverted their high *ERRB2* copy number to a near-diploid state and regained normal cell morphology. Third, PTs usually contain many additional aberrations that cannot be found in the UMs from the same breast. Fourth, aberrations detected in the UMs were typically also found in the PT(s) from the same breast but in a considerably higher proportion of cells. Fifth, we frequently observed that aberrations were found in samples collected at considerable distances (up to 24 cm), away from the focus of PT (Fig. 4). Sixth, we observed cases where UMs derived from different quadrants (i.e., likely from different breast lobes) were aberrant. In summary, our combined data set does not support the conclusion drawn by Sadanandam et al. (2012). Our data are, rather, compatible with a hypothesis that microscopically normal but genetically aberrant cells predate tumor cells and that clonal evolution of cells with early aberrations occurs, first arising in normal breast cells that subsequently evolve into tumor cells.

Breast-conserving surgery (also called lumpectomy, or sector resection) is a well-accepted standard of SCB patient care, and there is an ongoing debate regarding optimal resection margins that are sufficient to ensure radical removal of all cancer cells (Sadanandam et al. 2012). Our study provides evidence for presence of genetically altered cells in UM(s), sometimes located at unexpectedly large distances from PT(s). These cells, when left behind after a seemingly radical breast-conserving surgical intervention, may represent the source of local recurrence. These cells may also be responsible for distinct foci in multifocal or diffusely growing breast carcinoma. This issue clearly requires further studies. Our genome analysis of UM specimens pinpoints a number of well-known cancer genes which, when affected by the acquired copy number changes, can predispose to SBC. It should be stressed that gains are the dominating type of change in UMs with <105 Mb of total aberration load and likely represent early, driving events in the disease process. Our findings of frequent gains of *ERBB2* and five other cell membrane-bound receptor genes (*EGFR*, *FGFR1*, *IGF1R*, *NGFR*, and *LIFR*) might be important for the future improvements in diagnostics and therapy for SBC. For instance, Trastuzumab/Herceptin is an example of a drug that was developed to target *ERBB2*/HER2 and similar approaches are being taken for other receptors. Moreover, the knowledge of genes that are up-regulated via increased gene dosage (e.g., chromosomal gains) and encode proteins located on the cell surface of breast cells can be used toward development of new early diagnostic tests using modern imaging techniques, such as single-photon emission computed tomography (SPECT) or positron emission tomography (PET) (Mortimer et al. 2014; Sorensen et al. 2014). We hypothesize that such tests could detect an ongoing disease process much earlier (years, possibly even decades) compared to mammography. Medical treatment of cancer, including SBC, is currently focused on detection of primary tumors and management of advanced disease. Early detection of signals suggesting development of cancer, long before radiologically detectable tumors are formed, is a key aspect to the anticipated shift into a more preventive paradigm of personalized medicine. An extension of our results presented here offers a path in this direction.

## Methods

### Scheme of sample collection from participating centers and DNA isolation

The female breast cancer patients studied here were treated with radical mastectomy in four oncology centers in Krakow, Bydgoszcz, and Gdansk in Poland as well as in Falun, Sweden; 282 women with an initial diagnosis of SBC, regardless of age, were included in the study. The median age for all SBC patients was 60.2, range = 27–94. The criterion for inclusion was the availability of (1) primary tumor (PT) tissue; (2) matching control tissue (either blood or skin); (3) at least one sufficiently large sample (allowing genetic and histologic analysis) of macroscopically uninvolved margin (UM) specimen, that is, tumor-free breast tissue; and (4) >2 µg of DNA isolated from each of the above-mentioned samples, including at least one successful extraction of DNA from a UM sample. These latter samples were challenging during the DNA extraction procedure, because of the high content of fat tissue (see below) (Pekar et al. 2015). The clinical details of all studied subjects, including information about the type and number of samples studied by histopathology and genetic methods for each case, are shown in Supplemental Table 1. This study was approved by the Ethical Review Boards in Uppsala, Sweden as well as in Krakow, Bydgoszcz, and Gdansk, Poland.

The cohort from Bydgoszcz, Poland was the first analyzed in the course of this study. It was collected in 2004 and was partially reported previously during comparative analysis of primary tumors and lymph node metastases (Poplawski et al. 2010). From the Bydgoszcz cohort, we studied 80 UMs from 72 distinct cases with an age range of 27–79 yr (median = 60 yr). Typically one UM specimen (occasionally two) was available for each case that was excised at a distance of 4–8 cm away from the primary tumor. Most of the cases were diagnosed as unifocal disease (64 vs. seven multifocal cases).

In the Falun, Sweden cohort, a total of 256 UMs from 54 patients was studied. The diagnosis age span was 36–94 (median = 58 yr). The majority of these cases were multifocal (43 vs. 11 unifocal cases). Overall, 2–3 UMs surrounding each primary tumor were collected. Histopathology of all cases from Falun was studied using large-format histology slides of breast tissue (Figs. 5–7; Supplemental Fig. 18), which allows precise measurement of distances between UMs and PTs. An additional 1–2 UMs were taken for each case from parts of breast tissue as far away as possible from the segment (lobe) affected by breast cancer, which are labeled as UM98 and UM99. On average, 4.5 UMs were collected for each case in the Falun cohort. Control tissues for each case and 120 distinct PT samples were studied.

In the Krakow, Poland cohort, tissue samples from 146 subjects, aged 31 to 87 yr (median age at diagnosis = 62 yr), were included, providing a total of 805 UMs. The protocol of sample collection from this center as well as the Gdansk cohort (see below) is described in Supplemental Figure 19. The majority of these cases were unifocal (129), and 16 were multifocal. A mean number of five UM samples were collected for each case. The Gdansk, Poland collection of samples consists of 30 UMs from 10 cases included in the study. This cohort consists of women diagnosed with cancer at the age of 48–80 yr (median = 56 yr). Seven cases were unifocal, two were multifocal, and one was unclassified.

We have also examined 48 samples of normal breast tissue derived from reduction mammoplasty specimens of women without any suspicion or diagnosis of breast cancer from Uppsala, Sweden. These subjects were distributed in three age groups: 16 women with mean age of 25.3, 16 women with mean age of 39.2, and older women with mean age of 57.8. The median age of all the above control females was 40.8, range = 19–60.

The tissues were stored at −70°C prior to DNA extraction. The solid tissues were homogenized with a Tissuerupter (Qiagen). Proteinase K and sarcosine were then added, and the sample was incubated at 50°C overnight. The samples were transferred to Phase Lock Gel tubes, and the DNA was purified with phenol/chloroform extraction. Due to a very rich content of fat in UMs, phenol/chloroform extraction was repeated six times for all UMs and three times for PTs and control samples from skin. The purified DNA was precipitated with sodium acetate, pH 5.4 and 95% ethanol. The DNA precipitate was dried before dissolving in water. Control samples of blood were extracted with a QIAmp DNA Blood Maxi kit (Qiagen).

### Genotyping using Illumina and NimbleGen arrays as well as whole-genome next-generation sequencing

Successful SNP-array genotyping experiments were performed on control tissues (blood and/or skin), on one or more UMs, and PT samples from 282 breast cancer cases. The majority of genotyping experiments were conducted at the SNP&SEQ Technology Platform in Uppsala by using four similar in SNP-density Illumina platforms (Human 660W-Quad, Human1M-Duo, HumanOmniExpress, and HumanOmniExpressExome BeadChips), according to the recommendations of the manufacturer. An additional 48 Illumina genomic profiles of extra control samples were obtained by genotyping DNA extracted from healthy breast tissues from women who had undergone breast reduction surgery at the Uppsala University Hospital. The experiments were performed at the HudsonAlpha Institute for Biotechnology (Huntsville, AL), and the platform used was the Illumina HumanOmniExpress BeadChip. Validations were performed in 21 experiments from nine distinct SBCs using array-based comparative genomic hybridization on the NimbleGen 720K platform (Roche NimbleGen). These experiments were performed at the Medical University of Gdansk. The DNA of the control tissue (blood or skin) was cohybridized with the test DNA, derived either from UM or PT from the same case, as described previously (Forsberg et al. 2012). The unaveraged, normalized raw data were analyzed using Nexus 7.0 with the SNP-FASST segmentation algorithm and default settings.

The genotyping data were analyzed by Nexus Copy Number software version 7.0 (BioDiscovery) and passed a strict quality control (SNP call rate for all samples was >98%; the LogRdev value was <0.2) as previously described (Razzaghian et al. 2010; Forsberg et al. 2012, 2014). Log R ratio and B allele frequency values were imported into Nexus Copy Number, and copy number and allelic imbalances calls were made using the SNP-FASST segmentation algorithm with the following settings: significance threshold: 5.0 × 10^−9^; max contiguous probe spacing 500–1000; min. number of probes per segment: 10; homozygous frequency threshold: 0.85; homozygous value threshold: 0.95; and heterozygous imbalance threshold: 0.46. All the other parameters were left as default. After manual inspection of each copy number variant called by the Nexus software, and in order to compare the genomic profile of PT(s) with the aberrations observed in the UM tissue from the same subject, additional genotyping experiments were performed on one or more PTs from 157 subjects.

DNA obtained from nine additional samples (eight breast cancer cases and one healthy tissue control from mammoplasty) derived from laser-microdissection experiments (see below) was genotyped on the Illumina HumanOmniExpressExome platform at the SNP&SEQ Technology Platform in Uppsala. Experiments on this material showed an overall quality comparable with those performed on DNA extracted from fresh tissue. All the structural variants detected, along with all the phenotype information available for each case, were used to populate a MySQL database. Structural variants were summarized and visualized for various combinations of subjects using Circos plots (Krzywinski et al. 2009).

Structural variants from an additional two cases (four experiments) were validated with next-generation sequencing (NGS) performed at the HudsonAlpha Institute for Biotechnology (Huntsville, AL, USA), with an average coverage of 8–10×. These whole-genome profiles were analyzed using FREEC-software (Boeva et al. 2012), with default settings on sliding windows of 5 kb and after GC-content read-count normalization. FREEC converts sequence depth values into log R ratio pseudoprobes, which allows comparing these experiments with copy number calls from the Illumina SNP arrays.

### Validations of ERBB2 gain in normal breast cells using HER2 Dual ISH DNA Probe Cocktail Assay

Eleven cases from the Falun cohort were selected for validation using this method, based on the results of *ERBB2* analysis in UM samples from the Illumina platform. PTs from all these subjects were characterized as either HER2-positive or Luminal B HER2-positive, as part of routine clinical assessment. From large-format paraffin blocks, several tissue cores were removed, sectioned, and stained using the HER2 tricolor Dual ISH DNA Probe Cocktail Assay, according to the standard protocol (Roche Diagnostics Scandinavia AB) (Nitta et al. 2012). These tissue cores were selected based on their proximity to the original UM samples that were taken prior to fixation of the whole breast tissue (Figs. 5–7; Supplemental Fig. 18). *ERBB2* gene status was determined by detection of gene copies via silver in situ hybridization (SISH) and Chromosome 17 (Chr 17) copies via chromogenic red in situ hybridization (red ISH). This allowed us to see both overexpression of the HER2 protein (brown staining) as well as the copy number variation of two loci on Chr 17.

### Validation of genetic aberrations using laser-microdissection

Eight UMs and one sample of normal tissue from breast size-reduction surgery were analyzed by LMD, based on the availability of a sufficient amount of frozen UM tissue. We have also predominantly chosen cases with one, or a few, genetic aberrations scored in UMs from Illumina analyses. Initially, multiple thin (4–5 µm) sections were stained using hematoxylin and eosin, and this histological analysis further guided the decision about validation of a sample by this method. Frozen UM samples were further sectioned in 16- to 20-µm sections in a cryotostat with a chamber temperature of −35°C. The sections were placed on a FrameSlide with PET-membrane 1.4 µm (Leica Microsystems). The sections were then stained with cresyl violet staining (Sigma) according to a published protocol (Aaltonen et al. 2011), adding one extra step of an extended acetone wash at the end of the procedure, eliminating the excess of fat, which would otherwise make dissection difficult. The frozen sections were then examined for presence of histologically normal structures such as ducts and terminal ductal lobular units. These structures were then dissected out using a LMD6000 microscope (Leica Microsystems) with a UV-laser. Microscope settings LMD6000 during microdissection were as follows: laser power, max (60); aperture, 25; speed, 10; specimen balance, 25. The dissected pieces of tissue were then immediately collected in standard cell-lysis buffer containing a detergent. The highly dehydrated tissue was then rehydrated for at least 3 h at 50°C. Proteinase K was then added, and the DNA was extracted using a standard phenol/chloroform protocol. The extraction procedure was performed so that at least 200,000 microscopically normal epithelial cells were collected, thus avoiding an additional DNA amplification step. The LMD-extracted tissue DNA was then genotyped on the same platform as the original bulk DNA sample (HumanOmniExpressExome 950K), and the derived genomic profiles were compared.

## Data access

The complete set of Illumina SNP genotying data has been submitted to the NCBI Gene Expression Omnibus (GEO; http://www.ncbi.nlm.nih.gov/geo/) under accession number GSE64732. NimbleGen array data are available in the Supplemental material and also with the next-generation sequencing validation data at https://export.uppmax.uu.se/b2012110/Forsberg2015/.

## Competing interest statement

J.P.D. and L.A.F. are cofounders and shareholders in Cray Innovation AB. L.A.F. and J.P.D. are filing a patent application at The United Kingdom Intellectual Property Office to protect the commercial applications arising from the work in this publication.
